# Extranodal marginal zone lymphoma clonotypes are detectable prior to eMZL diagnosis in tissue biopsies and peripheral blood of Sjögren’s syndrome patients through immunogenetics

**DOI:** 10.3389/fonc.2023.1130686

**Published:** 2023-03-23

**Authors:** P. Martijn Kolijn, Erika Huijser, M. Javad Wahadat, Cornelia G. van Helden-Meeuwsen, Paul L. A. van Daele, Zana Brkic, Jos Rijntjes, Konnie M. Hebeda, Patricia J. T. A. Groenen, Marjan A. Versnel, Rogier M. Thurlings, Anton W. Langerak

**Affiliations:** ^1^ Department of Immunology, Laboratory Medical Immunology, Erasmus MC, Rotterdam, Netherlands; ^2^ Department of Immunology, Erasmus MC, Rotterdam, Netherlands; ^3^ Department of Paediatric Rheumatology, Sophia Children’s Hospital, Erasmus MC, Rotterdam, Netherlands; ^4^ Department of Internal Medicine, Division of Clinical Immunology, Erasmus MC, Rotterdam, Netherlands; ^5^ Department of Pathology, Radboudumc, Nijmegen, Netherlands; ^6^ Department of Rheumatology, Radboudumc, Nijmegen, Netherlands

**Keywords:** immunogenetics, Sjögren’s syndrome, early detection, lymphoma, lymphomagenesis

## Abstract

**Introduction:**

Activated B cells play a key role in the pathogenesis of primary Sjögren’s syndrome (pSS) through the production of autoantibodies and the development of ectopic germinal centers in the salivary glands and other affected sites. Around 5-10% of pSS patients develop B-cell lymphoma, usually extranodal marginal zone lymphomas (eMZL) of the mucosa-associated lymphoid tissue (MALT). The aim of the current study is to investigate if the eMZL clonotype is detectable in prediagnostic blood and tissue biopsies of pSS patients.

**Methods/Results:**

We studied prediagnostic tissue biopsies of three pSS patients diagnosed with eMZL and four pSS controls through immunoglobulin (IG) gene repertoire sequencing. In all three cases, we observed the eMZL clonotype in prediagnostic tissue biopsies. Among controls, we observed transient elevation of clonotypes in two pSS patients. To evaluate if eMZL clonotypes may also be detected in the circulation, we sequenced a peripheral blood mononuclear cell (PBMC) sample drawn at eMZL diagnosis and two years prior to eMZL relapse in two pSS patients. The eMZL clonotype was detected in the peripheral blood prior to diagnosis in both cases. Next, we selected three pSS patients who developed eMZL lymphoma and five additional pSS patients who remained lymphoma-free. We sequenced the IG heavy chain (IGH) gene repertoire in PBMC samples taken a median of three years before eMZL diagnosis. In two out of three eMZL patients, the dominant clonotype in the prediagnostic PBMC samples matched the eMZL clonotype in the diagnostic biopsy. The eMZL clonotypes observed consisted of stereotypic IGHV gene combinations (IGHV1-69/IGHJ4 and IGHV4-59/IGHJ5) associated with rheumatoid factor activity, a previously reported feature of eMZL in pSS.

**Discussion:**

In conclusion, our results indicate that eMZL clonotypes in pSS patients are detectable prior to overt eMZL diagnosis in both tissue biopsies and peripheral blood through immunogenetic sequencing, paving the way for the development of improved methods of early detection of eMZL.

## Introduction

Primary Sjögren’s syndrome (pSS) is a systemic autoimmune disease, characterized by impaired secretion of exocrine glands. Activated B cells play a key role in the pathogenesis of pSS, through the production of autoantibodies and the development of ectopic germinal centers in the salivary glands and other affected sites (1, 2). The produced autoantibodies cover a wide spectrum, including antinuclear, anti-Ro/SS-A, anti-La/SS-B and rheumatoid factor (RF) antibodies (3). In addition, 5-10% of pSS patients develop B-cell lymphoma, typically extranodal marginal zone lymphomas (eMZL) of the mucosa-associated lymphoid tissue (MALT), of which around 70% in the salivary glands (4). Lymphoma is the main cause of a decreased survival in pSS (5). Strikingly, parotid eMZL in pSS frequently express antibodies with RF activity, suggesting autoreactivity is an early driver during lymphoma development (6, 7). RF clones were previously shown to be enriched in inflamed tissues, particularly during pSS-related eMZL development (6). Therefore, a putative model for parotid eMZL lymphomagenesis has been proposed where RF clones organize in ectopic germinal center-like structures in a salivary gland, stimulating somatic hypermutation, proliferation and accumulation of driver mutations (8–10).

During their care for patients with pSS clinicians are confronted with a number of dilemmas. Patients are monitored for development of a complicated disease course by rheumatologists or immunologists, with lymphoma considered to be one of the most severe complications (11). The time to development of lymphoma varies with the highest incidence occurring after >8 years follow-up (12). Nonetheless, 50-70% of patients do not develop a complicated disease course, resulting in unnecessary costs for specialist care and anxiety for patients. Another dilemma occurs in the event of a clinical suspicion of lymphoma in patients with persistent general swelling of salivary glands or lymph nodes, or an unexplained mass lesion in other organs. A confident histological diagnosis can be difficult to make as eMZL in pSS develops in a background of chronic inflammation. This can result in uncertain circumstances in which a clear diagnosis cannot be reached, especially in small biopsies. Mortality and morbidity is increased in older patients with co-morbidities, with stage III/IV disease and in those with progression into a more aggressive lymphoma (11). Additionally, lymphoma treatment depends on staging and varies from wait-and-see policy, immunotherapy alone or mild chemotherapy in early stages to intensive chemo-immunotherapy in late stages and progression to diffuse large B-cell lymphoma.

Previously reported risk factors for lymphoma development in pSS include a combination of epidemiological, clinical, laboratory and pathological features. These prognostic markers include male gender, permanent parotid enlargement, lymphadenopathy, mixed monoclonal cryoglobulinemia, leukopenia, RF autoantibodies, low complement levels and an extensive lymphocytic infiltrate in a salivary gland biopsy (termed a high focus score) (4, 11, 13, 14). Some of the other risk factors may emerge as a reflection of pre-lymphomatous conditions, particularly parotid enlargement and cryoglobulinemia. Nonetheless, the specificity and sensitivity of these risk factors for lymphoma development is limited, and these features are not restricted to pSS patients developing eMZL. A composite score of lymphoma biomarkers improved specificity at the cost of sensitivity (71.8% sensitivity and 79% specificity at a score of ≥2) (4, 15). Unfortunately, the lack of clarity resulting from the limited specificity and sensitivity of existing markers can result in a significant diagnostic delay for eMZL diagnosis in pSS patients in the order of months to years, alongside a monitoring burden for pSS patients who never progress to eMZL. Altogether, there is a clear need for novel methods for early detection in pSS patients with high risk features for lymphoma.

Previously, we have shown that chronic lymphocytic leukemia (CLL), an indolent subtype of lymphoma, can be detected over 16 years prior to clinical diagnosis by sequencing the B-cell receptor immunoglobulin (BCR IG) gene repertoire in the peripheral blood (16). Our findings showed promise for early detection of lymphoma and for the use of immunogenetic sequencing for precision oncology. We hypothesize that patient groups at increased risk of lymphoma (such as pSS patients) in particular would benefit from a sensitive and specific method of early detection through immunogenetic sequencing.

Hence, the aims of the current study on pSS patients are: 1) to investigate the presence of the eMZL clonotype in tissue biopsies taken prior to eMZL diagnosis. 2) to perform an unbiased study of IG heavy chain (IGH) gene repertoire dynamics in the peripheral blood prior to eMZL and 3) to validate the presence of a dominant clonotype using the eMZL clonotype in the eMZL diagnostic biopsy.

## Material and methods

### Patient selection and sampling

We selected three pSS cases with prediagnostic tissue biopsies dating up to seven years before eMZL diagnosis and 4 pSS controls who did not develop eMZL and sequenced the immunoglobulin (IG) gene repertoire in all tissues. The selected tissue biopsies originated either from the moment of pSS diagnosis (parotid or labial biopsies) or from biopsies taken due to unexplained symptoms, such as a swollen lymph node or parotid gland. Tissue type of diagnostic eMZL biopsy varied based on dissemination of eMZL at diagnosis (parotid, lymph node, liver, bronchial or breast). We then explored the technical feasibility of detection of the eMZL clonotype in the circulation by sequencing the peripheral blood of two pSS patients who had developed eMZL, either at the time of lymphoma diagnosis or 2.3 years prior to eMZL relapse. For the retrospective study on early detection of eMZL in the peripheral blood, we designed a case-control study. We selected three pSS patients who were diagnosed with eMZL and five pSS patient controls matched for age, sex and blood sample availability. All peripheral blood mononuclear cell (PBMC) samples were drawn after pSS diagnosis and prior to eMZL diagnosis. On average, disease duration was longer for controls (12-25 years) than for cases (6-8 years) at time of sampling. A total of sixteen longitudinal PBMC samples (seven from cases, nine from controls) were included in the study, drawn at a median of three years before eMZL diagnosis (interquartile range two years). For two of the three pSS patients with prediagnostic PBMC samples who developed eMZL, a matched diagnostic tissue biopsy was available. A schematic overview of the study design can be found in **Supplementary Figure 1**. Biopsies were stored in fresh frozen (FF) or formalin fixed and paraffin embedded (FFPE) form. Collection and usage of samples was approved by the IRB of both Erasmus MC and Radboudumc (MEC2011-116; MEC2019-484; MEC2015-1721) and studies were performed in accordance with the Declaration of Helsinki.

### DNA isolation and immunogenetic sequencing

Genomic DNA was isolated from tissue biopsies using the GenElute™ Mammalian Genomic DNA Miniprep Kit (Sigma-Aldrich, St. Louis, MO) or from PBMCs using the GenElute™ Blood Genomic DNA Kit (Sigma-Aldrich). A leader-based PCR was utilized to amplify the IGH repertoire of the PBMC samples and the FF tissue biopsies (17). 500 ng of gDNA was given as input for the multiplex PCR for the PBMC samples and 20-40 ng of gDNA for the tissue biopsies. The PCR product was sequenced on the Illumina Miseq platform. For the FFPE samples, an adapted protocol was used to account for fragmentation resulting from DNA crosslinking. DNA from FFPE samples was sequenced using IG clonality protocol through Illumina sequencing (18). The sequencing data were then annotated through the ARResT/Interrogate immunoprofiler (19). A clonotype was defined as a rearrangement with an identical HCDR3 amino acid sequence using the same IGHV-gene and IGHJ-gene. Clonotypes utilizing an IGHV-gene and IGHJ gene combination associated with RF stereotypy (IGHV4-59/IGHJ2, IGHV4-59/IGHJ5, IGHV1-69/IGHJ4, IGHV3-7/IGHJ3) were considered to present with RF-like features. None of the clonotypes with RF-like features in the current study were an identical match to RF clonotypes from literature. A quality control table for the sequencing results can be found in the supplement (**Supplementary Table 1**).

### Data analysis

During data analysis, the abundance of the largest (“dominant”) clonotype was contrasted to the background clonotypes by calculating a ratio, i.e. by dividing the abundance of the dominant clonotype by the mean of the abundance of the clonotypes ranked 3^rd^-7^th^, as previously reported (20). Subsequently, the ratio was compared between cases and matched controls. Dominant clonotypes identified in the diagnostic biopsies were considered to reflect the malignant clonotype and were tracked back in the prediagnostic IGH repertoire in the peripheral blood. Intraclonal diversification was evaluated using IgIDivA after processing the data through the T cell receptor/immunoglobulin profiler TRIP (21, 22).

## Results

### eMZL clonotypes are detectable in tissue biopsies of pSS patients before eMZL diagnosis

**Figure 1:**
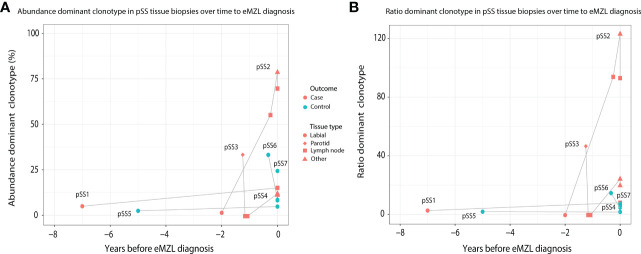
Abundance of eMZL clonotype in tissue biopsies obtained from pSS patients up to eMZL development. **(A)** The abundance of the dominant clone as a percentage of total reads in the IG gene repertoire (IGH for pSS1 and pSS2, IGK-Kde for pSS3, IGH for pSS4-7) is shown in various tissue samples of SS patients developing eMZL and pSS controls. **(B)** The ratio of the abundance of the dominant clonotype clonotype compared to the clonotypes ranked 3rd-7^th^ is depicted for both cases and controls. Tissue biopsies are further described in **Supplementary Table 2**.

In order to investigate the prediagnostic presence of eMZL clonotypes, we sequenced prediagnostic tissue biopsies of three pSS patients developing eMZL and four pSS controls who did not develop eMZL. We detected the eMZL clonotype in prediagnostic tissue biopsies for all three pSS patients (**Figure 1**; **Supplementary Table 2**). For two patients (pSS1 and pSS2), the eMZL clonotype was detected in the IGH gene repertoire, while for the last patient (pSS3) we detected it in the immunoglobulin kappa-deleting element (IGK-Kde) repertoire, a rearrangement well recognized as a clonal marker in lymphoma (23, 24). In prediagnostic tissue biopsies where the eMZL clonotype was present, the eMZL clonotype was observed with abundances ranging from 4.2%-55% for all patients (**Figure 1A**). Clonal proliferations exceeding 10% were also detected for two out of four controls, pSS6 and pSS7, though the clonal proliferations did not remain present in the follow up biopsy for pSS6, suggesting dominant clonal proliferations in pSS patients who do not develop eMZL may be transient in nature. Determination of the abundance and longevity of clonal proliferations in pSS patients is essential to evaluate the potential for early detection of eMZL through immunogenetic sequencing. Calculating the ratio of the abundance of the eMZL clonotype compared to the clonotypes ranked 3^rd^-7^th^ preserved longitudinal dynamics and appears to enhance differentiation between cases and controls in the current cohort, as previously described for other lymphoma subtypes, though our sample size is insufficient to establish a meaningful cutoff (**Figure 1B**) (20). In all 3 cases, the dominant clonotype observed in the earlier tissue biopsy matched the clonotype observed at eMZL diagnosis (**Supplementary Table 2**). Notably, for patient pSS3, the eMZL clonotype was only observed in the parotid gland biopsy, while two prediagnostic lymph node biopsies taken several months later showed no dominant clonotype, indicating limited dissemination of the eMZL clonotype. Interestingly, both patients with a detectable prediagnostic eMZL clonotype in the IGH gene repertoire (pSS1 and pSS2) presented with RF-like features (IGHV1-69/IGHJ4), while none of the dominant clonal proliferations in tissue biopsies of controls presented with RF-like features. The dominant eMZL clonotype was detected up to 7 years before diagnosis for patient pSS1.

### The eMZL clonotype is detectable in the peripheral blood at diagnosis and prior to relapse

**Figure 2:**
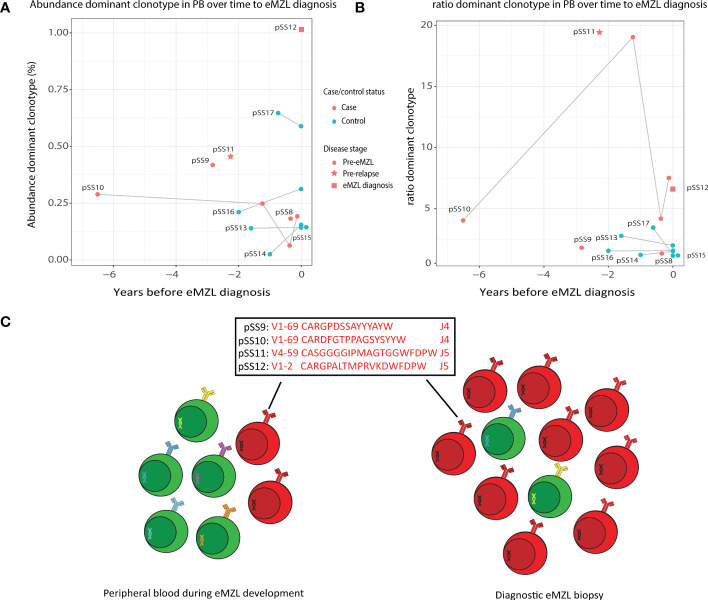
Abundance of eMZL clonotype in peripheral blood of pSS patients. **(A)** The abundance (% of total reads in the IG gene repertoire) of the eMZL clonotype in pSS patients is shown over time until eMZL diagnosis in comparison to matched pSS controls. For controls, the abundance of the dominant clonotype is shown. **(B)** The ratio (#1 clonotype divided by the mean of clonotype #3-#7) in pSS patients is shown over time to eMZL diagnosis in comparison to matched pSS controls. PBMC samples and matching diagnostic biopsies are further described in **Supplementary Table 3**. **(C)** Schematic overview of early detection of premalignant eMZL clonotypes. The eMZL clonotypes identified in both tissue biopsy and the peripheral blood in the current study are highlighted.

To evaluate the potential for a less invasive approach of early detection of eMZL using peripheral blood of pSS patients, we first sequenced the IGH gene repertoire of PBMC samples of two pSS patients (pSS11 and pSS12) diagnosed with eMZL, in patient pSS12 at the time of eMZL diagnosis and for patient pSS11 2.3 years prior to eMZL relapse. In both patients, we observed skewing of the IGH gene repertoire in the peripheral blood in both abundance and ratio (**Figures 2A, B**), though the skewing was more pronounced for the ratio. We additionally sequenced diagnostic tissue biopsies for these patients. For the patient with the relapsed eMZL (pSS11), the dominant clonotype identified in the peripheral blood 2.3 years prior to relapse was also an exact match to the malignant clonotype identified at relapse. For the sample drawn at diagnosis of eMZL (pSS12), the 2^nd^ ranked clonotype matched the diagnostic eMZL clonotype (**Supplementary Table 3**), while the 1^st^ ranked clonotype did not. Neither the 1^st^ ranked clonotype nor the eMZL clonotype displayed RF stereotypy in patient pSS12. In summary, our data indicates that the detection of the eMZL clonotype is feasible in the peripheral blood at eMZL diagnosis and prior to relapse.

### The eMZL clonotype is detectable in the peripheral blood prior to diagnosis of eMZL

Based on these results, we hypothesized that the eMZL clonotype may be detected in the circulating cells in the blood prior to diagnosis as well. Therefore, we designed a retrospective case-control pilot study screening the peripheral blood of pSS patients prior to eMZL diagnosis. For two patients (pSS9 and pSS10) a matched diagnostic biopsy at eMZL diagnosis was available, allowing identification of the eMZL clonotype. In both cases, the dominant clonotype in the prediagnostic blood sample matched the eMZL clonotype (**Supplementary Table 3**). These eMZL clonotypes consisted of IGHV/IGHJ rearrangements with RF-like features (IGHV1-69/IGHJ4 and IGHV4-59/IGHJ5). Furthermore, the eMZL clonotype observed in one of these two patients (pSS10) was already present seven years prior to eMZL diagnosis in the peripheral blood (**Figures 2A–C**). For the third pSS patient developing eMZL lymphoma (pSS8), no overt prediagnostic skewing was detected whilst the actual eMZL clonotype could not be determined due to the lack of availability of a diagnostic biopsy.

### The absolute abundance of the eMZL clonotype in the peripheral blood does not significantly differ from the controls but the ratio of the dominant clonotype over the clonotypes ranked 3^rd^-7^th^ is increased among pSS cases at eMZL diagnosis

The absolute abundance of the eMZL clonotypes in the peripheral blood remained relatively low, at less than 1% of the total IGH gene repertoire and no significant difference was observed in the absolute abundance of the dominant clonotype of cases and controls (**Figure 2A**). This observation indicates a fundamental difference between prediagnostic tissue biopsies in pSS patients *vs*. the peripheral blood. In prediagnostic tissue biopsies where the eMZL clonotype was present, a dominant clonal proliferation could be observed with abundances ranging from 4.2%-55% for all patients (**Figure 1**; **Supplementary Table 2**). Interestingly, similarly to the eMZL clonotypes observed at eMZL diagnosis and prior to relapse in patients pSS11 and pSS12, the eMZL clonotypes were the highest ranked (most abundant) clonotypes present in the peripheral blood of patients pSS9 and pSS10 prior to eMZL diagnosis. To quantify the magnitude of the increase in relative abundance, we calculated a ratio between the abundance of the dominant clonotype in a sample divided by the mean abundance of the clonotypes ranked 3^rd^-7^th^ (20). We observed a marked increased abundance of the eMZL clonotype compared to the clonotypes ranked 3^rd^ to 7^th^ at diagnosis of eMZL and prior to eMZL diagnosis in patients pSS10, pSS11 and pSS12 (**Figure 2B**). Thus, the peripheral blood IGH gene repertoire of pSS patients developing lymphoma may not be characterized by a pronounced increase in the absolute abundance of the clonotype of interest, as previously observed for CLL (16), but instead by an increase in the ratio or relative abundance of the eMZL clonotype in comparison to the mean of the abundance of the clonotypes ranked 3^rd^-7^th^ (**Figures 2A, B**).

### Abundant and persistent clonotypes with RF-like features may have potential as a marker for eMZL development in pSS patients in tissue biopsies and peripheral blood

**Figure 3:**
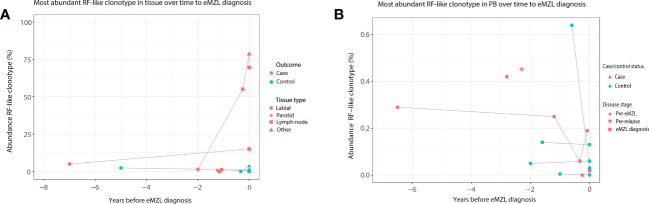
Abundance of RF-like clonotypes in cases and controls in tissue and peripheral blood of pSS patients. **(A)** The abundance (% of total reads in the IG gene repertoire) of the most abundant RF-like clonotype in pSS patients is shown over time until eMZL diagnosis in tissue biopsies in comparison to matched pSS controls. **(B)** The abundance (% of total reads in the IG gene repertoire) of the most abundant RF-like clonotype in pSS patients is shown over time until eMZL diagnosis in the peripheral blood in comparison to matched pSS controls.

Based on these interesting findings, we then evaluated whether the abundance of clonotypes with RF-like features (IGHV1-69/IGHJ4, IGHV4-59/IGHJ2, IGHV4-59/IGHJ5 or IGHV3-7/IGHJ3) may have additional potential as a marker for eMZL development. In tissue biopsies, elevation of clonotypes with RF-like features was only observed for pSS patients developing eMZL (2 out of 3 cases), suggesting highly abundant clonotypes with RF-like features may indeed have potential as a marker for eMZL development (**Figure 3A**). In circulation, RF-like clonotypes were observed in both cases and controls, albeit at low abundance in comparison to the tissue biopsies (**Figure 3B**). Interestingly, persistent presence of the same RF-like clonotype as the dominant clonotype (and matching the eMZL clonotype) was a feature restricted to pSS patients developing eMZL, while controls presented with transient elevation of RF-like clonotypes only (**Figure 3B**). The utility of detection of clonotypes with RF-like features in the peripheral blood for the early detection of eMZL remains unclear, although it would be of interest to explore if an aberrant RF-like clonotype identified in tissue biopsy might be monitored through the peripheral blood as a less invasive alternative.

### Intraclonal diversification is limited in the peripheral blood compared to the intraclonal diversification in the eMZL clonotype in the diagnostic biopsy and SHM levels are variable in the peripheral blood over time to diagnosis

**Figure 4:**
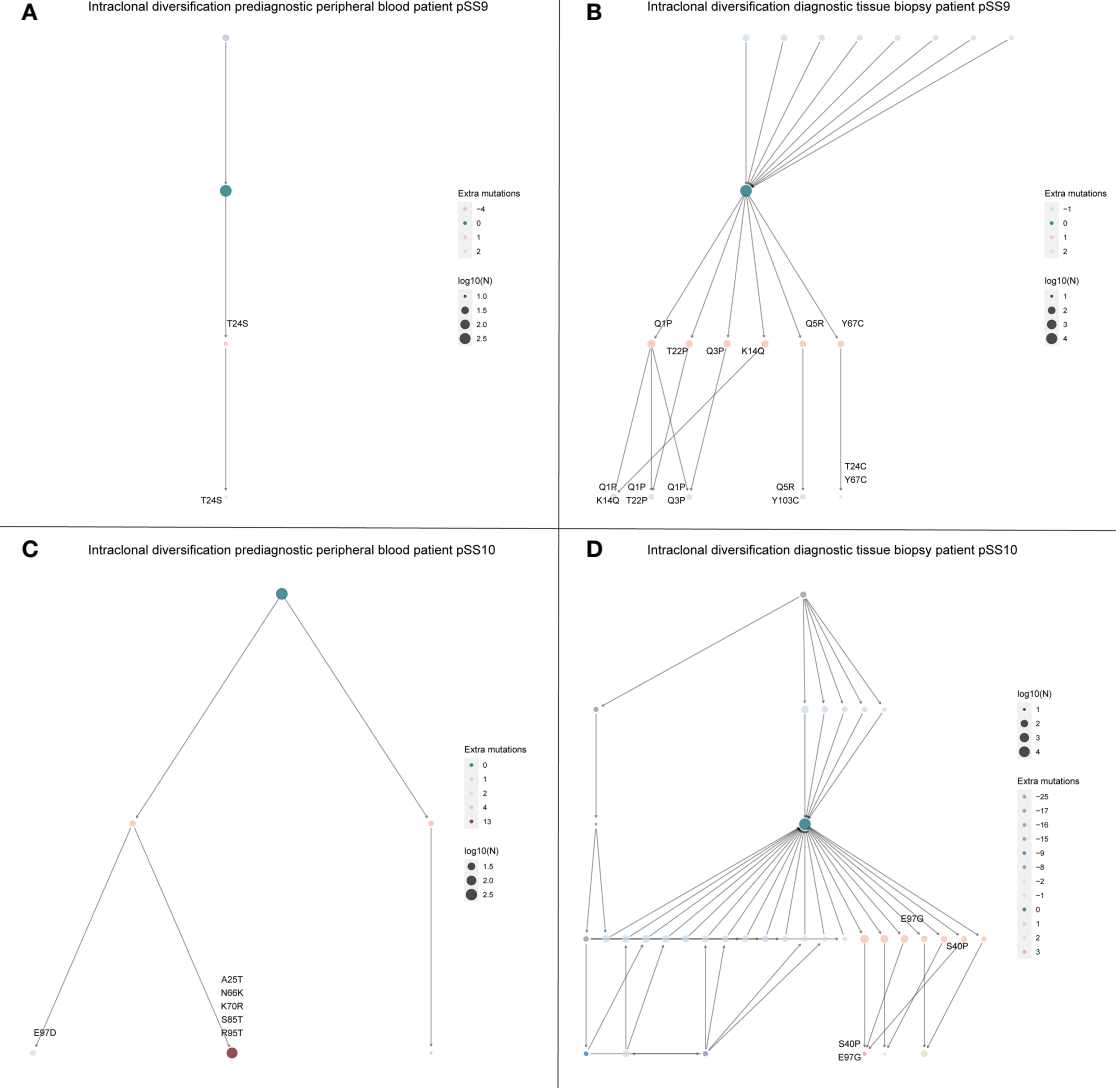
Intraclonal diversification of the eMZL clonotype is strongly reduced in the peripheral blood *vs.* the diagnostic tissue material. The intraclonal diversification (ID) of the eMZL clonotype in pSS patients is shown for two patients with prediagnostic PBMC samples available. For these two patients a fresh frozen tissue biopsy was available at eMZL diagnosis. Subclones with reduced mutational load are indicated as precursors, while subclones with additional mutations are indicated as diversification within the eMZL clonotype. **(A)** ID pattern in patient pSS9 in the peripheral blood prior to eMZL diagnosis **(B)** ID pattern in the diagnostic tissue biopsy of patient pSS9. **(C)** ID pattern in patient pSS10 in the peripheral blood prior to eMZL diagnosis. **(D)** ID pattern in patient pSS10 in the diagnostic tissue biopsy of patient pSS10. Log10(N) refers to the number of sequencing reads assigned to each subclone and is used to scale the size of each subclone.

Intraclonal diversification as a consequence of high levels of somatic hypermutation is a well described feature of eMZL lymphomas in pSS (6). In the diagnostic tissue biopsies we indeed observed extensive intraclonal diversification (**Figures 4B, D**). Interestingly, we observed very limited intraclonal diversification within the eMZL clonotype in the peripheral blood (**Figures 4A, C**). SHM levels in the peripheral blood increased over time to diagnosis of eMZL, while SHM levels in the peripheral blood shortly before diagnosis and at diagnosis matched SHM levels in the diagnostic biopsy, suggesting ongoing SHM during eMZL development (**Supplementary Table 3**).

## Discussion

In this study, we sequenced the IG gene repertoire of tissue biopsies and/or the peripheral blood of eight pSS patients who developed eMZL and nine matched pSS controls. Our results indicate eMZL clonotypes are already present in both tissue biopsies and the peripheral blood prior to eMZL diagnosis. In pSS patients, dendritic cells, T-cells and B-cells react strongly to auto-antigens such as the ribonucleoprotein particles Ro/SS-A and La/SS-B expressed by the epithelium of the exocrine glands. They produce high levels of cytokines and chemokines, resulting in chronic inflammation of the exocrine glands and loss of physiological function (25, 26). The salivary gland epithelium plays a central role in this local autoimmune process by actively stimulating immune cells to accumulate, activate and differentiate (27). The inflammatory microenvironment and activated immune cells then create a vicious cycle by activating the epithelial cells and promoting epithelial cell survival, resulting in a perpetual maintenance and escalation of pSS-associated auto-immune responses (27). A particularly important factor in the context of eMZL development is that epithelial cells may be directly involved in B-cell activation. Salivary gland epithelial cells of pSS patients have been shown to produce BAFF mRNA upon Type 1 IFN stimulation (28). BAFF is essential in promoting B lymphocyte activation and survival and has also been associated with autoimmune B-cell activation (27, 29). B-cells in pSS can be found in focal mononuclear infiltrates and between the ductal epithelial cells in the salivary glands (30, 31). Interestingly, the B-cells found in the focal mononuclear infiltrates express markers associated with memory B-cells and plasma cells, while the B-cells in the lympho-epithelial lesions between the ductal epithelial cells express markers of chronic activation and proliferation in absence of classical memory and plasma cell markers (30, 31). This population of chronically expanding B-cells has been described as the putative source of the eMZLs associated with pSS (32, 33).

One of the most impactful and most studied pathways in pSS is the Type 1 interferon (IFN) pathway (34). The IFN receptor IFNAR is expressed on nearly all cells in the body and its downstream mediators affect the transcription of up to 10 percent of all human genes (35, 36). A remarkable and consistent upregulation of IFN stimulated genes (commonly referred to as the IFN signature) has been reported in pSS (37, 38). A higher expression of IFN stimulated genes is associated with a more severe disease phenotype characterized by B-cell hyperactivity, manifesting through a higher focus score, autoantibody seropositivity, hypergammaglobulinemia, complement consumption, reduced saliva secretion and an increased risk of lymphoma development (39).

The susceptibility of RF clones for lymphomagenesis compared to anti-Ro/La clones has been attributed to differences in affinity maturation. The increased incidence of lymphomagenesis among RF clones likely results from differences in the structure of the recognized autoantigens. Compared to anti-Ro/La clones, RF clones express a restricted public set of immunoglobulin variable regions and an IgM constant region, resulting in extensive intraclonal diversification, which may over time result in formation of cryoglobulins and monoclonal lymphoproliferation (6, 8). In the current study, we observed evidence for this intraclonal diversification in the diagnostic tissue biopsies, but not in the eMZL clonotype in the peripheral blood. Additionally, the degree of intraclonal diversification in the eMZL clonotype in the peripheral blood was stable over time to diagnosis (data not shown). This observation would suggest that intraclonal diversification may hold value as a marker for eMZL development in the context of tissue biopsies but not in the peripheral blood, although this finding should be validated in a larger study.

In the context of this chronic auto-immune inflammatory process, it is perhaps not surprising that malignant B-cell clonotypes are already detectable prior to eMZL diagnosis. It remains unclear at what point after the emergence of these clonotypes progression to eMZL occurs and what drivers are involved in progression. To answer this question it will be essential to ascertain which (genetic) driver events lead to the transformation from auto-immune RF-stereotyped B cells to eMZL and which role the local environment of the parotid gland epithelium plays in this process. Further study of the features of the prediagnostic IG gene repertoire may be an effective method of providing clarification on the dynamics of the expansion during eMZL development, but paired experiments evaluating the local environment will likely be required to achieve new insights. Additionally, in the event of the identification of a clonal population in the initial salivary gland biopsy during pSS diagnosis it may be beneficial to monitor the putative eMZL clonotype in the peripheral blood instead, reducing the burden of additional biopsies required for monitoring. Another factor to consider is whether there is a difference in the sensitivity of eMZL detection through immunogenetics when sequencing DNA from labial salivary gland biopsies *vs.* parotid gland biopsies.

The detection of a Kde clonotype alone without an IGH rearrangement in the tissue biopsy of one pSS patient (pSS3) highlights the benefit of immunogenetic sequencing beyond the IGH gene repertoire, as in this instance the malignant clonotype would have been missed. Potentially, the extensive degree of somatic hypermutation observed in eMZL has impeded amplification of the IGH rearrangement in this patient (24). The IGK-Kde is a IGK rearrangement in which recombination occurs of a recombination signal sequence 24 kb downstream of IGKC gene with a IGKV gene or with the intron between the IGKJ and IGKC genes. In lymphoma, this rearrangement is well recognized to be used as a clonal marker (23, 24).

RFs present with stereotypic combinations of IGHV and IGHJ genes with shared CDR3 sequence motifs (32, 40, 41). Interestingly, the stereotypy seen in RFs is shared with parotid eMZL developed by pSS patients (7, 32, 40). These eMZLs express one of a specific set of stereotyped combinations: IGHV4-59/IGHJ2, IGHV4-59/IGHJ5, IGHV1-69/IGHJ4, IGHV3-7/IGHJ3 (40). These IGH rearrangements are usually accompanied by a IGK light chain rearrangement involving the IGKV3-15 or IGKV3-20 gene (40). In the current study, five out of eight pSS patients developed eMZL expressing IGHV1-69/IGHJ4 or IGHV4-59/IGHJ5 rearrangements, supporting their putative value as prediagnostic markers for eMZL. Not all eMZL in pSS patients present with RF-like features, as described previously. Additionally, we observed a clonotype with RF-like features (IGHV3-7/IGHJ3) in one control (pSS13) at a ratio of 2.8 (**Supplementary Table 3**). This clonotype could still be detected 1.6 years later, though it was no longer the clonotype with the highest abundance in the repertoire. Another control (pSS17) presented with a clonotype with RF-like features (IGHV1-69/IGHJ4) at a ratio of 3.5. This clonotype had significantly diminished 7 months later (6-fold reduction in abundance). Therefore, a combination of factors should be considered before characterizing a clonotype as potentially (pre)malignant. These include RF-like stereotypic features, consistent elevation of a clonotype over time and the magnitude of the increase in abundance and/or ratio. Characterization of a clonotype as potentially malignant based on measurement in the circulation alone appears prone to false positives. An interesting potential application of our findings would be to perform routine immunogenetic sequencing of the parotid or labial gland biopsy taken at pSS diagnosis. In the event that a (RF-like) clonotype is detected at an abnormal abundance, this clonotype could then be monitored through the peripheral blood, rather than through repeated biopsies, significantly reducing the monitoring burden for the patient, while putatively detecting eMZL at an earlier stage. Integration of immunogenetic sequencing based risk factors with previously reported risk factors for lymphoma in pSS such as parotid enlargement, cryoglobulinemia, leukopenia, detection of rheumatoid factor autoantibodies and low complement levels may further improve risk stratification (4). In our cohort, pSS patients developing eMZL were characterized by a combination of intermittent parotid enlargement, cryoglobulinemic vasculitis, high focal score (≥4) (13), anti-Ro, anti-La and low complement at time of sampling (seven years to three months before eMZL diagnosis), while most of these risk factors (except anti-Ro) were not reported in the controls (**Supplementary Table 4**).

The main limitation of the current pilot study is its sample size, preventing further investigation into heterogeneous features of both the lymphomas and included pSS patients. The distinct prediagnostic dynamics of the eMZL clonotypes observed in both the tissue biopsies and the peripheral blood indicate a larger cohort is needed to fully understand the implications of the observed prediagnostic eMZL clonotypes, both for our understanding of lymphomagenesis in pSS patients and to evaluate the potential of these clonotypes for the early detection of eMZL.

In conclusion, eMZL clonotypes can be detected prior to eMZL diagnosis in both tissue biopsies and in the peripheral blood of pSS patients. The context-dependent abnormalities we observed in the IGH gene repertoire of blood and tissues in pSS patients have potential implications for early detection of lymphoma risk. While the absolute abundance of the observed eMZL clonotypes in tissues and peripheral blood was not sufficient to readily distinguish them from clonotypic expansions in controls, features such as RF-stereotypy, ongoing SHM and a persistently increased abundance relative to the other clonotypes in the IGH gene repertoire have the potential to aid in risk stratification and warrant further investigation. Furthermore, our findings highlight the potential of pSS-associated RF clones to transform into eMZL, confirming previous reports. In the age of precision oncology, there is a need for high resolution data and insights on targeted patient groups. While our results suggest potential merit for early detection of eMZL development through immunogenetic sequencing, expansion of the cohort size will be an essential step in validating the intriguing findings from the current pilot study.

## Data availability statement

The datasets presented in this study can be found in online repositories. The names of the repository/repositories and accession number(s) can be found below: https://www.ncbi.nlm.nih.gov/, GSE221412.

## Ethics statement

The studies involving human participants were reviewed and approved by Medical Ethics Review Committee Erasmus MC and Central Committee on Research Involving Human Subjects, Netherlands. The patients/participants provided their written informed consent to participate in this study. Written informed consent was obtained from the individual(s) for the publication of any potentially identifiable images or data included in this article.

## Author contributions

PMK and JR performed the experiments. PMK and PJTAG analyzed the data. PMK, EH, MJW, CGH-M, PLAD, ZB, JR, KMH, PJTAG, MAV, RMT and AWL interpreted results. PMK and AQL wrote the manuscript. EH, MJW, CGH-M, PLAD, ZB, JR, KMH, PJTAG, MAV, RMT critically reviewed and edited the manuscript. MAV, RMT and AWL designed and supervised the study. All authors contributed to the article and approved the submitted version.
