# Supplementary figures and images for: Extranodal marginal zone lymphoma clonotypes are detectable prior to eMZL diagnosis in tissue biopsies and peripheral blood of Sjögren’s syndrome patients through immunogenetics
Source: Front Oncol. 2023 Mar 23;13:1130686. doi: 10.3389/fonc.2023.1130686 (PMC10076775; doi:10.3389/fonc.2023.1130686)

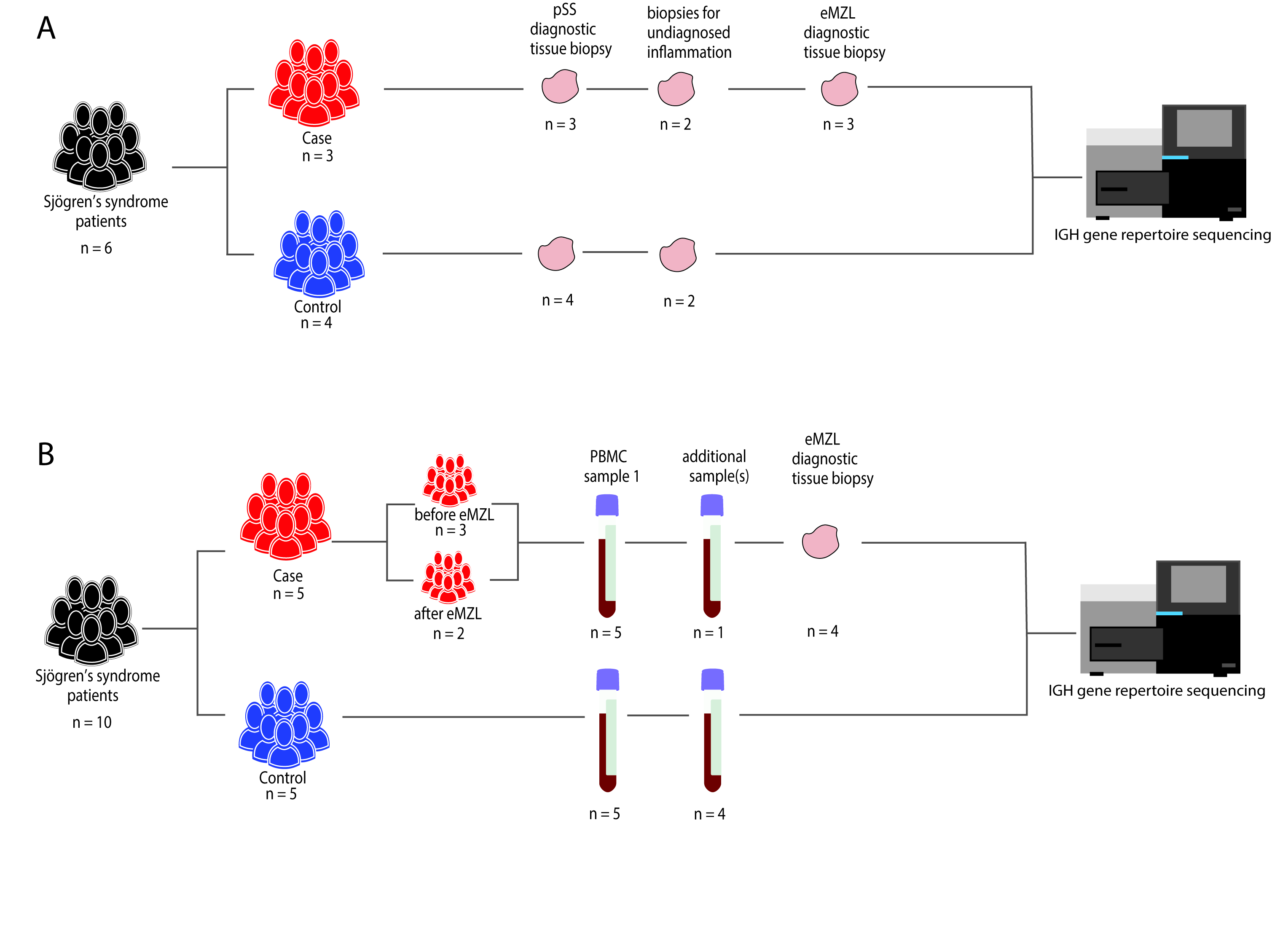

Supplement: Supplementary file 1 [file Image_1.tif]
